# Conservation of HIV-1 T cell epitopes across time and clades: validation of immunogenic HLA-A2 epitopes selected for the GAIA HIV vaccine

**DOI:** 10.1186/1742-4690-9-S2-P294

**Published:** 2012-09-13

**Authors:** AS De Groot, K Sangare, O Koita, M Ardito, C Boyle, L Levitz, J Rozenhal, K Tounkara, S Dao, Y Kone, Z Koty, L Moise, W Martin

**Affiliations:** 1 EpiVax, Inc, Providence, RI, USA; 2 University of Bamako, Bamako, Mali; 3 GAIA Vaccine Foundation, Providence, RI, USA; 4 GAIA Foundation, Bamako, Mali; 5 Infectious Disease and Tropical Medicine Unit Point G, Bamako, Mali

## Background

HIV genomic sequence variability has complicated efforts to generate an effective globally relevant vaccine. Our strategy for HIV-1 vaccine design is to select epitopes that can induce broad and dominant HLA-restricted immune responses targeted to the regions of the viral genome conserved in sequence, three-dimensional configuration, and across time which may represent regions that are constrained due to functional or structural limitations. These “Achilles’ Heel” epitopes would be ideal candidates for inclusion in an epitope-based HIV vaccine.

## Methods

Highly conserved T-cell epitopes were selected using the EpiMatrix suite of immunoinformatic tools. This analysis was first performed in 2002 on 10,803 HIV-1 sequences available at that time and again in 2009 on an expanded 43,822 sequences. Selected epitopes were validated for binding and immunogenicity with PBMCs from HIV-infected donors in Providence, RI and Bamako, Mali.

## Results

38 highly conserved HLA-A2 candidate epitopes were selected. Analysis done in 2009 revealed surprising stability of 25 of the epitopes selected in 2002 and identified an additional 13 highly conserved HLA-A2 candidates. Thirty-five (92%) of the 38 selected epitopes stimulated IFNg response in PBMC from at least one subject. Twenty-one of 25 peptides selected in 2002 were validated in assays performed in Providence. Eleven (85%) of the 13 peptides selected in 2009 were confirmed in assays performed in Mali. Twelve of 18 peptides assayed in both Providence and Mali were confirmed in both locations.

## Conclusion

The validation of these selected HLA-A2 epitopes conserved across time (2002-to-2009), geography (Providence and Mali) and clades supports the hypothesis that these epitopes could provide effective coverage of virus diversity and would be appropriate candidates for inclusion in a globally relevant HIV vaccine.

